# Supplementary material for: Resolving the full spectrum of human genome variation using Linked-Reads
Source: Genome Res. 2019 Apr;29(4):635–45. doi: 10.1101/gr.234443.118 (PMC6442396; doi:10.1101/gr.234443.118)
Supplement: Supplemental Material [file supp_gr.234443.118_Supplemental_longranger-2.2.zip › longranger-2.2.tar.bz2!/lr2.2-public/lib/stan/cmdstan/stan/doc/api/html/common_2sample_8hpp_source.html]

Stan: src/stan/common/sample.hpp Source File


|  |  |
| --- | --- |
|  | Stan  2.5.0  probability, sampling & optimization |


- Main Page
- Related Pages
- Namespaces
- Classes
- Files

- File List
- File Members

All Classes Namespaces Files Functions Variables Typedefs Enumerations Enumerator Friends Macros Pages

- src
- stan
- common

sample.hpp

Go to the documentation of this file.

1 #ifndef STAN\_\_COMMON\_\_SAMPLE\_HPP

2 #define STAN\_\_COMMON\_\_SAMPLE\_HPP

3

4 #include <stan/mcmc/base\_mcmc.hpp>

5 #include <stan/io/mcmc\_writer.hpp>

6 #include <stan/common/run\_markov\_chain.hpp>

7

8 namespace stan {

9  namespace common {

10

11  template <class Model, class RNG, class StartTransitionCallback,

12  class SampleRecorder, class DiagnosticRecorder, class MessageRecorder>

13  void sample(stan::mcmc::base\_mcmc\* sampler,

14  int num\_warmup,

15  int num\_samples,

16  int num\_thin,

17  int refresh,

18  bool save,

19  stan::io::mcmc\_writer

20  <Model,SampleRecorder,DiagnosticRecorder,MessageRecorder>& writer,

21  stan::mcmc::sample& init\_s,

22  Model& model,

23  RNG& base\_rng,

24  const std::string& prefix,

25  const std::string& suffix,

26  std::ostream& o,

27  StartTransitionCallback& callback) {

28  run\_markov\_chain<Model, RNG, StartTransitionCallback,

29  SampleRecorder, DiagnosticRecorder, MessageRecorder>

30  (sampler, num\_samples, num\_warmup,

31  num\_warmup + num\_samples, num\_thin,

32  refresh, save, false,

33  writer,

34  init\_s, model, base\_rng,

35  prefix, suffix, o,

36  callback);

37  }

38

39  }

40 }

41

42 #endif

run\_markov\_chain.hpp

base\_mcmc.hpp

mcmc\_writer.hpp

stan::io::mcmc\_writer

mcmc\_writer writes out headers and samples

**Definition:** mcmc\_writer.hpp:29

stan::common::run\_markov\_chain

void run\_markov\_chain(stan::mcmc::base\_mcmc \*sampler, const int num\_iterations, const int start, const int finish, const int num\_thin, const int refresh, const bool save, const bool warmup, stan::io::mcmc\_writer< Model, SampleRecorder, DiagnosticRecorder, MessageRecorder > &writer, stan::mcmc::sample &init\_s, Model &model, RNG &base\_rng, const std::string &prefix, const std::string &suffix, std::ostream &o, StartTransitionCallback &callback)

**Definition:** run\_markov\_chain.hpp:13

stan::mcmc::sample

**Definition:** sample.hpp:13

stan::common::sample

void sample(stan::mcmc::base\_mcmc \*sampler, int num\_warmup, int num\_samples, int num\_thin, int refresh, bool save, stan::io::mcmc\_writer< Model, SampleRecorder, DiagnosticRecorder, MessageRecorder > &writer, stan::mcmc::sample &init\_s, Model &model, RNG &base\_rng, const std::string &prefix, const std::string &suffix, std::ostream &o, StartTransitionCallback &callback)

**Definition:** sample.hpp:13

stan::mcmc::base\_mcmc

**Definition:** base\_mcmc.hpp:13


---

[ Stan Home Page ]

*© 2011–2014,
Stan Development Team.*
